# The Effects of the COVID-19 Pandemic on Nutrition, Health and Environment in Indonesia: A Qualitative Investigation of Perspectives from Multi-Disciplinary Experts

**DOI:** 10.3390/ijerph191811575

**Published:** 2022-09-14

**Authors:** Oyedolapo A. Anyanwu, Elena N. Naumova, Virginia R. Chomitz, Fang Fang Zhang, Kenneth Chui, Martha I. Kartasurya, Sara C. Folta

**Affiliations:** 1 Friedman School of Nutrition Science and Policy, Tufts University, 150 Harrison Ave, Boston, MA 20111, USA; 2 Public Health & Community Medicine, School of Medicine, Tufts University, 136 Harrison Ave, Boston, MA 20111, USA; 3 Department of Public Health Nutrition, Faculty of Public Health, Universitas Diponegoro, Semarang 50275, Jawa Tengah, Indonesia

**Keywords:** COVID-19, diets, population health, marine environment, multi-disciplinary team

## Abstract

Objectives: The COVID-19 pandemic impacted food systems, health systems and the environment globally, with potentially greater negative effects in many lower-middle income countries (LMICs) including Indonesia. The purpose of this qualitative study was to investigate the potential impacts of the COVID-19 pandemic on diets, health and the marine environment in Indonesia, based on the perspectives of a multidisciplinary group of informants. Methods: We conducted remote in-depth interviews with 27 key informants from many regions of Indonesia, who are either healthcare providers, nutrition researchers or environmental researchers. Interview question guides were developed based on a socio-ecological framework. We analyzed the data using a qualitative content analysis approach. Results: Informants suggested that while the COVID-19 brought increased awareness about and adherence to good nutrition and health behaviors, the impact was transitory. Informants indicated that healthy food options became less affordable, due to job losses and reduced income, suggesting a likely increase in food insecurity and obesity. Environmental researchers described higher levels of marine pollution from increase in hygienic wastes as well as from plastic packaging from food orders. Conclusions: Our findings reveal perceptions by informants that the increased awareness and adherence to health behaviors observed during the pandemic was not sustained. Our results also suggest that the pandemic may have exacerbated the double-burden paradox and marine pollution in Indonesia. This study offers information for generating hypotheses for quantitative studies to corroborate our findings and inform policies and programs to mitigate the long-term impacts of the COVID-19 on diets, health, and the marine environment in Indonesia.

## 1. Introduction

In March 2020, the World Health Organization identified the novel coronavirus (SAR-CoV-2) the carrier of the Corona Virus 2019 (COVID-19) as a global pandemic and declared a global health emergency [1,2]. To date (June 2022), a total of 540 million cases and 6.3 million deaths have been attributed to the COVID-19 pandemic globally [3]. There is increasing concern about the long-term impacts of the pandemic on health risk factors for lower-middle income countries (LMICs) such as Indonesia, who already had high disease burdens and inadequate health infrastructure, prior to the outbreak of the pandemic [4,5,6].

Since the global outbreak of the COVID-19 pandemic, Indonesia has experienced three major waves. The first wave was between March to September 2020, the second wave was between June to September 2021 and the most current from January to March 2022 [7]. Following the recommendations from the WHO, to curb the spread of the virus and flatten the curve, the Indonesian government implemented Large-Scale Social Restrictions (LSSR) policies, which included closing workplaces, schools, public transportation, and socio-cultural activities in public places from March ending 2020 to September 2020 [7,8]. However, similar to other countries, social restrictions had adverse socio-economic impacts including reduced work force, job losses, mental health impacts, and sedentary behaviors [6,8]. Further, Indonesia reported the highest mortality figures from the COVID-19 among countries in the South East Asia region [5,8,9]. Table 1 shows the cumulative number of confirmed cases and deaths due to the COVID-19 as of June 2022. Indonesia not only had the highest number of deaths but also the lowest proportion of the population fully vaccinated in the region [3].

Prior to the COVID-19 pandemic, Indonesia had high rates of undernutrition and obesity coexisting side by side, an epidemiologic state referred to as the *double burden paradox*, which is commonly found in many LMICs going through nutrition transition [9,10,11,12]. Nutrition transition is characterized by marked changes in dietary patterns, usually towards unhealthy food options, that give rise to adverse health outcomes and significant public health crises [13,14,15].

Previous studies from Indonesia investigating the impacts of COVID-19 on nutritional outcomes focused on the period of COVID-19 restrictions, and used mainly online survey methods and cross-sectional designs, and have yielded inconclusive results [16,17]. Purnasari et al., reported an increase in hunger scale from 6% pre-pandemic to 11% during the active phase of the LSSR in Daerah Istimewa Yogyakarta (DIY), Indonesia and highlighted the need for further studies to address the long-term impacts of COVID-19 on food insecurity among vulnerable populations [16]. In a cross-sectional study to assess the impacts of COVID-19 on food security in urban and semi-urban areas in Jakarta, authors reported that COVID-19 negatively impacted food security of families in both urban and semi-urban areas equally, but noted the challenges of developing a culturally sensitive instrument through online surveys [17].

COVID-19 also exacerbated existing chronic health conditions [18], and people with pre-existing chronic conditions had a higher risk for contracting COVID-19 [19]. More so, with a high level of uncertainty about the COVID-19 virus among scientists and epidemiologists, no vaccine, and no effective drug therapy during the active phase of the pandemic, nutrition was considered a robust preventive approach [1,20,21,22,23]. However, the evidence on the benefits of nutrition interventions on overall health outcomes during the COVID-19 in Indonesia is mixed [1,22]. Fauziyana et al., surveyed diet quality of elderly respondents over 60 year from five sub-district health centers in urban areas of Jakarta (an epicenter of the COVID-19 pandemic in Indonesia), based on Healthy Eating Index (HEI) scores of a 2-day 24 h food recall [22]. A low score was reported for total fruit intake, whole grains and dairy, but excess intake beyond recommended values were found for refined grains and saturated fats [22]. Authors concluded prevalent low diet-quality among respondents and highlighted the need for further studies on COVID-19 impacts among the general population [22]. In a scoping review of different combinations of nutrition and physical activity interventions to manage metabolic syndrome [24] during COVID-19 in South East Asia, authors reported limited evidence for the effectiveness of these interventions on MetS, and called for more studies to address the gaps in knowledge, particularly on the effects of nutrition interventions on MetS [18]. 

Additionally, Indonesia being the world’s largest island country, has one of the highest levels of marine pollution in the world [25,26]. While fish and other marine products are major food types and important sources of key nutrients for the Indonesian population, consistent evidence has shown the detrimental impacts of marine pollution on fish quality and availability and ultimately on human health [27,28,29]. Since the COVID-19 pandemic, there is emerging evidence that pollution levels have substantially increased globally, and more specifically in Indonesian seas and oceans. In a study that used mathematical models to simulate the magnitude of the pandemic-related wastes in the marine environment of 193 countries, authors found that medical wastes made up 73% of the global waste load, and that 72% of this was from the Asia region [30]. Another cross-sectional study in Indonesia that investigated riverine contents before and after COVID-19 in Jakarta Bay showed a 5 percent increase in the abundance of debris compared to pre-COVID period, with an unusually high content of COVID-19 related medical wastes [31]. However, more evidence is needed to inform policy guidelines for marine waste management in Indonesia post-COVID-19 pandemic [31].

Our study will extend the current literature on the COVID-19 pandemic in Indonesia by synthesizing the perspectives from a muti-disciplinary group of stakeholders from the health sector and the marine environment, to help bridge the knowledge gap on the multifaceted and interrelated impacts of the pandemic on diets, health and the marine environment in Indonesia. Our findings will provide evidence to help inform policy guidelines and the design of health programs targeted at specific vulnerable populations.

## 2. Methods

### 2.1. Study Design and Conceptual Framework

**Figure 1:**
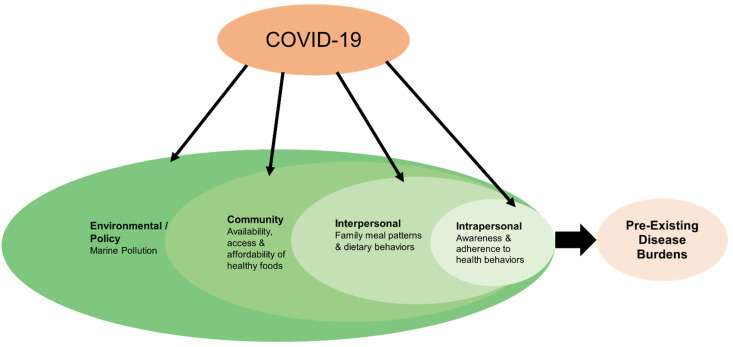
Socio-ecological model of COVID-19 impacts on dietary behaviors and disease risk factors in Indonesia.

This study used theory-based, qualitative methodology to understand perspectives of expert informants on the dietary behaviors of the Indonesian population, and how dietary behaviors might be impacting health outcomes. Here, we particularly focus on the effects of the COVID-19 pandemic on dietary, health and environmental factors. The socio-ecological model (SEM) was used as an organizing framework for the study (Figure 1). The SEM allows for a more holistic and contextual examination of health behaviors by identifying multiple levels of influence (intrapersonal, interpersonal, community, and global policies) on an individual’s health behavior [32]. Hence, in recent years, there is a shift from single-level, individual-focused behavior change theories to broad, comprehensive frameworks to investigate health behaviors [33,34,35]. Intrapersonal factors are factors within an individual such as their knowledge, attitudes and beliefs that inform their health behaviors. Within this level, interview questions were designed to assess the effects of the COVID-19 pandemic on health awareness and adherence among the Indonesian population. The interpersonal factors are external sources of influence on an individual’s health behaviors, such as from parents, peers, or significant others. Here, questions were designed to examine the influence of the COVID-19 pandemic on family meal patterns and dietary behaviors. The community and regional level factors address issues in the physical environment among those who share location and regional boundaries [33]. At this level, the questions were designed to investigate how the COVID-19 impacted the availability, affordability, and access to healthy food types for individual and families living in a particular community or region. The global and policy level factors are upstream factors that transcend what are commonly found across communities and regional boundaries that influence an individual’s health behaviors. At this level, questions were designed to help understand the impact of the COVID-19 pandemic on marine pollution. In addition to this theory-based approach, we also used an inductive approach, to offer interpretation of findings grounded in the data.

### 2.2. Description of Research Team

The study was conducted by a multidisciplinary team of Ph.D. level academic researchers from Tufts University, Boston MA and Universitas Diponegoro, Semarang Indonesia, who have many years of teaching and research experience (ENN, VRC, FFZ, KC, MIK and SCF), and one doctoral student (OAA), who coordinated the study as part of her doctoral dissertation project. The research team combined expertise from the fields of nutrition, epidemiology, public health and environmental health. The senior author of the study (SCF) is a qualitative expert with extensive publications using the qualitative approach. All but one of the research team members are of the female gender.

### 2.3. Location and Recruitment

The first author (OAA) and a trained research assistant conducted in-depth interviews online with a purposive sample of key informants from across Indonesia (*n* = 27), who are either healthcare providers, public health nutrition researchers or environmental researchers, between April and June 2021, a period when Indonesia had just emerged from the first wave of the COVID-19 pandemic and when the LSSR policies were starting to ease. Informants’ responses therefore reflect their perspectives on the different topics addressed before the pandemic, during the LSSR, after the LSSR and before the second wave. One participant was unable to be interviewed due to COVID-19 infection. The sample size was adequate to capture a range of perspectives and reached saturation on the impacts of the COVID-19 on the topics addressed at the different levels of the framework. We determined that we have reached saturation when no new information or perspective was shared that had not been previously expressed. Recruitment followed a snowball recruitment strategy. Two members of the study team (ENN and MIK) recommended the first set of informants that were recruited and interviewed. We then asked these informants to provide additional contacts at the end of their interview sessions. A proficiency in the English language was a criterion to participate in the study. Invitations to participate in the study were sent by emails and those who respond to the emails were sent a link to fill out a demographic questionnaire. Healthcare providers were recruited based on their familiarity with the dietary needs and behaviors of the adult population in Indonesia. Public health nutrition researchers were recruited for their expertise in food environment and nutrition policy in Indonesia. Environmental researchers were selected for their expertise on the impacts of environmental pollution on the quality and availability of heart-healthy foods such as fish, fruits, and vegetables. A lack of internet access was an exclusion criterion since the interviews were conducted online. A study team member read the informed consent document to each participant by WhatsApp audio call and received their verbal consent prior to each interview session. The study was approved by the institutional review boards (IRB) of Tufts University and the Universitas Diponegoro (UNDIP) Indonesia.

### 2.4. In-Depth Interviews Procedures

Based on the SEM framework as described above, a semi-structured interview guide was developed by a multidisciplinary team consisting of experts in the field of qualitative methods and epidemiology, as well as two collaborators who are public health nutritionists from UNDIP in Indonesia. Study collaborators from UNDIP reviewed the question guide for cultural relevance. We then pilot-tested with two Indonesians in relevant areas of expertise, who share similar demographic characteristics with those we recruited for the study, to ensure that the questions on the guides were interpreted as intended. We iteratively refined the interview guide as the study progressed by eliminating and streamlining questions as needed, for better clarity. The final guide is provided in Table 2. From the pilot interviews, we found that while informants had a lot to say, it took them a long time to express their perspectives in English, since English is not the official language of Indonesia, even though they can understand and have adequate fluency in it. To address this problem and avoid unnecessarily long interview sessions, under advisement from Indonesian study collaborators, we sent the question guide ahead of time to informants so they could prepare their responses. Before the start of each interview session, the interviewer reminded the informant of the purpose of the study and their rights as an informant and explained that the research was part of a doctoral dissertation project. Each interview session lasted between 45 to 60 min and was recorded. In addition, the interviewer took notes of the non-verbal responses of the informant. Only the interviewer and informant were present during each session. At the end of each session, the interviewer provided a general summary to validate perceptions of the main points.

### 2.5. Data Analysis

Recorded data from the in-depth interviews were transcribed verbatim. Transcripts were crosschecked against the audio recordings and notes taken during the sessions. A codebook was developed iteratively from the transcripts, using a qualitative content analysis approach. The analysis was primarily deductive based on the study framework, although some themes emerged inductively. To establish intercoder reliability, two study team members independently coded three randomly selected transcripts, one from each category of experts, using the codebook. No major issues were identified in code agreement except the need to better clarify the description of some codes. Once the minor discrepancies were addressed, the intercoder reliability score was >90% agreement and at least 0.75 on the kappa coefficient for all codes. With the codebook refined and performing well, we proceeded with the full coding of all the transcripts. The NVivo software (QSR International Pty Ltd. (2018) NVivo (Version 12), Burlington, MA 01808, USA) was used to create codes and assist with analysis. In the deductive analysis phase, we closely explored each code to understand the ideas being conveyed by participants by running word searches and text queries. We conducted multiple rounds of coding to more meaningfully categorize and combine ideas, thoughts and views. For the inductive analysis, we developed codes for salient ideas that emerged from the data that did not fit into the framework. We followed the same iterative process of exploration and categorization of codes as was conducted at the deductive phase. Categories that reflect similar ideas were combined as themes, and topics addressed under each theme were classified as sub-themes. Emerging themes were iteratively reviewed and refined and later verified by study collaborators in Indonesia [36].

## 3. Results

### Description of Study Informants

Table 3 shows the demographic breakdown of study informants. The majority were women (70.4%). Their mean age was 46 years (SD 11.9). The ethnicity breakdown was predominantly Javanese (52%). About 48% of the informants hold a PhD degree, 22% have a professional medical or nutritional qualification, 18% hold a Master’s degree, and the remaining 11% have a Bachelor’s degree. When categorized by years of working experience, a greater proportion of informants who are nutrition and environmental researchers had at least 11 years working experience, while the majority of those who are healthcare providers had worked less than 5 years.

## 4. Themes

Informants described the double-burden in Indonesia that existed prior to the pandemic. In that context, four themes emerged around changes due to the pandemic across the four levels of the socio-ecological framework. At the individual level, one theme emerged on the impact of COVID-19 on awareness about and adherence to health behaviors. The family/interpersonal level revealed one theme on the impact of COVID-19 on family meal patterns and dietary behavior. At the community/regional level, one broad theme on the impact of COVID-19 on the food environment emerged, which was categorized into three sub-themes addressing the impact of the pandemic on availability, affordability, and access to healthy food types. Finally, at the environmental/policy level, one theme emerged relating to the impact of COVID-19 on marine pollution. Table 4 summarizes key findings on the impacts of the COVID-19 across the four levels of the SEM framework, based on the perspective of our informants.

### 4.1. Theme: COVID-19 Impacts on Awareness about and Adherence to Health Behaviors

Informants described how the COVID-19 pandemic affected general awareness about and adherence to health behaviors, including diets and physical activity among individuals. Through campaigns by the government on social media and health education by healthcare providers, tastes and preferences that typically drive food choices were no longer the primary factor as people prioritized foods that are associated with enhancing immunity. This led to higher demand for animal proteins, fruits and vegetables as well as spices, herbs and supplements.

*“COVID-19, I think the one that I previously mentioned because they concern about increasing immunity. So, for example, household that maybe never, because I noticed also in my family the household that never eat fruits or vegetables, and now they eat it once a day. There is a change like that and they consume vitamin supplement as well and herbal. Herbals like spices.”*

(44-year-old female, a public health nutrition researcher).

*“Yes, prior to COVID, they prefer western food and fast food like that but after COVID, because there are so many education in the locations and also in social media, so most people are trying to change their habit and prefer to choose fruit first, before... besides the fatty foods.”*

(56-year-old female, a public health nutrition researcher).

Informants described more people taking up some form of physical activity during and after the active phase of the LSSR through gardening, jogging or cycling. However, they also noted that sedentary behaviors generally increased, particularly during the period of restrictions in mobility.

*“In the COVID-19 era in the urban people, now, so many exercise activity like small group, for example, riding bike together with friend or jogging together with the kids. One good thing from the COVID-19 is changing the exercise activity, especially for the urban people.”*

(38-year-old female, a healthcare provider).

*“After COVID, I’ve seen lots of people taking up cycling. Even some healthcare practitioners that I work with, they’ve also become more interested in cycling.”*

(IDQ 18, 22-year-old female, a healthcare provider).

*“Exercise habit was decreased. Why exercise has decreased? Our people like to exercise in the public area. Right now, some and many public area was closed because of the COVID.”*

(54-year-old female, a public health nutrition researcher).

Informants reported that while COVID-19 might have exacerbated many health programs due to restrictions in movements, it also facilitated the adoption of online health consultations such as telemedicine services. This was considered with mixed feelings however, because although access to online health consultation was beneficial, there was also an avalanche of health information on social media from both authentic and non-authentic sources.

*“Yes. Because we have phone, they sometimes look for the recommendation from the internet because they have mobile phone like that and it’s easy to get. They can get information easier than before but sometimes because so many organizations or so many people give information, sometimes they get confused where is the right information because so many information, so many recommendation for people, everyone can give recommendation like that.”*

(36-year-old female, a healthcare provider).

Informants pointed out that although COVID-19 brought increased awareness about and adherence to health behaviors, the behavior change was not sustained as society gradually went back to normal once the restrictions were lifted.

*“I think there’s nothing changed with the COVID. I think COVID had huge impact only in a few months after it’s announced. Everything’s getting back to normal”*

(44-year-old male, an environmental researcher).

*“Not changed, I guess [laughs]. Based on my experience, if I talk to my patients, they still buy foods, deep frying food from street vendors, because it already become our habit eating behavior, a habit. So it’s really hard not to buy deep frying foods from street vendors, because here in Indonesia we can find the street vendors everywhere… and… it’s part of our habit.”*

(33-year-old female, a healthcare provider).

### 4.2. Theme: COVID-19 Impacts on Family Meal Patterns and Dietary Behaviors

Informants explained that with restrictions on mobility in place in many regions, many restaurants and fast-food shops had to close temporarily and many went out of business altogether. Additionally, due to the growing health awareness and fear of contracting the virus, more families were taking their meals at home. For urban centers, these may be meals ordered from outside and eaten at home or meals cooked at home or a combination of both, depending on the time constraints, particularly for working women, who still had to work remotely.

*“… in my area I noticed that before COVID-19, maybe every day the restaurants always full. Sometimes pizza or other local food restaurants always full, but now many of them even already closed. It means, it can indicate that people may be tend to eat more frequent at home, or they can order online and then they still eat at home but buying online.”*

(44-year-old female, a public health nutrition researcher).

*“I don’t think the habit has really changed much except for, I heard that more people are starting to cook more at home, things like that. For me, since I work in Jakarta full time, I still don’t have time to cook. I usually buy around a place. I don’t do that cooking myself. Lots of people, I heard they start to do more cooking themselves.”*

(22-year-old female, a healthcare provider).

For people in rural areas and families with a low income, they would be doing more home cooking. But this is due more to economic reasons than to health concerns, since many will qualify to receive food aid, usually basic staples and essentials like rice, meat, cooking oils, etc., either from the government or from other charitable organizations.

*“With lower income, in my region, there are some jobless people… the government help this people, so the government give like nutrition packet: Supplements, and then some foods and fruits for these people.”*

(29-year-old male, a healthcare provider).

*“what I observe depends very much on the socioeconomic level. Because, you know, it is now quite difficult time from economic points of view, so they will cook, rather than buying.”*

(69-year-old female, a public health nutrition researcher).

Once the LSSR policies were lifted, the demand for away from home foods rebounded to pre-COVID-19 levels, and probably surpassed it, because informants also described increase in food orders from online food platforms that were proliferating. Informants additionally noted the development of home catering services, where many families with cooking skills began taking food orders.

*“Based on my experience and what I see and what I know, there is not much difference…, because maybe, at the beginning of the COVID-19 they afraid to buy food from outside, from restaurants but I guess it only lasted just, maybe two or three months. But, nowadays, they’re not afraid anymore, … they still buy food from outside, even though they do not eat at the restaurant, they buy the food from the restaurant, or they order, the food from online delivery. So I guess there is not much difference about the food preference.”*

(33-year-old female, a healthcare provider).

*“No, before pandemic we don’t have a food service business, but I think this is a chance, the real chance during pandemic… we can sell foods… with low price, so can we help more people to eat good food… And then send happiness to the people. I think many many families within my area will have specialists or they have cooking abilities, they build this little industry like home industry for food service. This has become the common, the new common situation.”*

(29-year-old male, a healthcare provider).

Informants suggested that school closure and mothers restricting children from going outside to play meant more screen time and more exposure to food advertisements for children and adolescents, which led to increased demand for away from home foods.

*“I think it now has a bigger influence because parents prefer kids at home the whole day. They prefer rather than letting kids playing outside. They will open television or other programs from the internet. Of course, it changes and sometimes the kids don’t want to eat their mother’s food because they want to eat that food that they see on the television or the internet, so it happens also.”*

(38-year-old female, a healthcare provider).

### 4.3. Theme: COVID-19 Impacts on Availability, Affordability and Access to Healthy Foods

Informants elaborated on how COVID-19 impacted food availability, affordability, and access.

### 4.4. Sub-Theme: COVID-19 Impacts on Availability of Healthy Foods

Informants described panic purchase by consumers at the early days of the pandemic which created temporary shortage of essential food commodities, but they noted that supplies eventually stabilized again. They noted that while disrupted supply chains from farmers to consumers affected the quantity and quality of perishable items, such as fruits and vegetables, shortage in supplies were supplemented by increase in home gardens and higher demand for frozen foods.

*“…Yes, in an early pandemic in March 2020 in [name of region] informed this is a pandemic scale so, the food is a lack. Many people panic buying and then many local governments give the local restrictions, we are full lockdown here, so the access of food is really terrible, only food stock from the refrigerator can serve us.”*

(28-year-old female, a public health nutrition researcher).

*“Then for those who cannot go outside to get fresh food because they are afraid of going to market because in the beginning, COVID market is the place of transmission, in Indonesia traditional market, especially, so they then use frozen food business that is mushrooming following that situation.”*

(44-year-old male, an environmental researcher).

Informants indicated that fish availability followed a similar trend of temporary shortage, similar to other healthy food types, due to fishermen not being able to go to fish during the active lockdown phase, but that it gradually returned to pre-COVID-19 levels.

*“Then it’s getting worse when we have a pandemic, COVID, because the distribution is really limited by our government. It’s make our fishermen, they just catch the fish near the areas, not for miles in open ocean. The fish they catch is more little than as usual.”*

(40-year-old female, an environmental researcher).

### 4.5. Sub-Theme: COVID-19 Impacts on Affordability of Health Foods

Informants demonstrated various ways that COVID-19 impacted the affordability of foods. Many people lost their jobs as a result of many businesses failing, which decreased their purchasing power. Prices of foods considered immunity-boosting e.g., meat, fruits, vegetables, spices and herbs, rose due to increasing demands for these items. Informants implied that decreased purchasing power and high cost of foods may have increased food insecurity and malnutrition.

*“…but in low income, especially in pandemic COVID, many people lost their jobs, and then they have little money for consuming a better food. Maybe, they always buy some cheap foods like carbohydrates but meat or protein is expensive, yeah.”*

(29-year-old male, a healthcare provider).

*“Maybe because of the COVID-19 impacted people’s income and people become difficult to buy food, and then the price of food in urban get increase, and then impact their supply from rural also. Rural area may become food insecure because they have to sell. They have to sell off all of their own products.”*

(44-year-old female, a public health nutrition researcher).

Although the government had food and monetary aid programs to support the poor, these were generally insufficient, and were often supplemented by food aid programs from charitable organizations. Moreover, many government and charitable programs to help the poor were adversely affected due to restrictions on mobility and people’s fear of contracting the virus.

*“Due to the affordability, I already mentioned that even we have a social safety net or money from the government to support the poor family, but if the market is closed, they cannot access. In order to meet their need or to buy the food they need, they will go to the small retail, in which the price is a little bit higher compared to the open market. It will influence the number of the food or the variety of the food that they afford to buy due to the increased price.”*

(45-year-old female, a public health nutrition researcher).

*“After this COVID-19 affected Indonesia, I think all over the world but especially in Indonesia, there’s so many program that cannot do well. Like health Indonesian insurance that have program that I told you before, so many programs cannot that they do because we have to aware about the protocol, we have so many things that we have to prepare to make the meeting. So many program we cannot do that.”*

(36-year-old female, a healthcare provider).

### 4.6. Sub-Theme: COVID-19 Impacts on Access to Healthy Foods

LSSR policies notwithstanding, informants felt that COVID-19 did not negatively impact access to healthy foods because with the proliferation of online food retail platforms people still had access to healthy foods. Moreover, while many people voluntarily reduced the frequency of going to places with a high transmission of the virus, for safety concerns, the ability to sell and buy food through online platforms still made it possible to access healthy foods.

*“I think, in my observation it doesn’t really change about access…, but what in the market it may a little bit different from before. Like, like now, they, they have like herbal more, but of course more expensive. Like that. But when you talk about access, I think this… that it doesn’t really change a lot, access yeah because, they, they are available, it’s just what you want to buy, so it depends very much … on economic status of the community—higher status gets more access”*

(69-year-old female, a public health nutrition researcher).

*“Maybe since COVID, there are so many of them, buy it from online, so they do not have to go to the market so they buy it online. COVID-19 pandemic not really gave impact in food access. People in low-income neighborhood still could access some food items. This pandemic impact on how they could buy it.”*

(54-year-old female, a public health nutrition researcher).

Additionally, informants implied that the proliferation of online platforms cut off the role of middle-men in the supply chain, which may have helped to drive down the price of some food items.

*“Well, it’s because it’s getting more expensive I think that because there are so many application that it’s called ‘Petani’. Petani means farmers that offering their product directly to the consumer which is really good so there is no in-between, which usually takes more profit than the farmers. If the farmers get a better price directly from consumer, then it’s good for them than before. COVID is not always negative actually, there is a blessing in disguise in this COVID.”*

(60-year-old female, a public health nutrition researcher).

*“Yes. Of course, yes. Traditional market and supermarket is so high-priced to COVID-19. Now, in my home, we have and seller, vegetable seller. We can order them by WhatsApp, and we can always with vegetable and fruit or anything. We can list, and they will buy to traditional market and send us. For me, it’s better choices. I must not go anywhere, but they can send me anything I need. I think like that.”*

(36-year-old female, a healthcare provider).

### 4.7. Theme: COVID-19 Impacts on Marine Pollution

Informants with expertise in environmental research described higher levels of marine pollution due to COVID-19. These were from increased volume of medical and hygienic wastes from masks and personal protective equipment [37], as well as from plastic packaging from online food orders that get dumped into the seas.

*“I have two students conduct this research, the briefly result of ours is during the pandemic, the solid waste increase, but the kind of waste is different with before. Before the pandemic, maybe plastic and organic material is very high concentrate in ocean, but now, the health waste, I mean the medical waste like masks or all of the medical waste is increased in the ocean. I know that medical waste is dangerous.”*

(45-year-old male, an environmental researcher).

*“With additional factors, the medical waste, as well as the hygiene-related waste. And more plastic for sure because of the COVID. We have more plastic. When you have online delivery service, be it for food or other stuff, you need more wrapping. You need more wrapping material, like plastics. As a result, we have more waste than normal.”*

(59-year-old male, an environmental researcher).

Environmental researchers also noted that government prioritizing the rising health needs due to COVID-19 meant less attention given to addressing marine pollution. Moreover, they reported that other community-led programs to reduce marine waste were also negatively impacted.

*“Because of the COVID-19, most of the budget goes to the health sector. I think it’s very difficult for the government to allocate enough financial support for the waste management and taking care of the marine pollution. It becomes like the number maybe 9 or 10 priority because I think the priority to the health and economic sector.”*

(45-year-old female, an environmental researcher).

*“Then also, the activity for material recovery is restricted or reduced because for example in Indonesia …where woman is picking up and collect plastic and other recyclable material, but during COVID-19, their activity is restricted and some of them is afraid. I believe that polluting is increasing.”*

(45-year-old male, an environmental researcher).

## 5. Discussion

This study sought to understand the multi-disciplinary perspectives of key informants from many regions of Indonesian on the impacts of the COVID-19 pandemic on population health and environment in Indonesia, through in-depth interviews conducted remotely. To the authors’ knowledge, this is the first study using a qualitative inquiry method to understand the multi-dimensional impacts of the COVID-19 pandemic on diets, health and marine pollution—three areas with major public health significance for Indonesia.

At the individual level, our analysis captured one major theme relating to awareness and adherence to health behaviors before and since the pandemic. Previous studies investigating the impacts of COVID-19 on diet and health awareness and adherence in Indonesia have uncovered mixed results. In a cross-sectional study to assess the awareness and adherence to the government imposed social restrictions among respondents 16 years and above in West Java during the month of June 2020, authors found high awareness about the pandemic, and that knowledge significantly correlated with healthy behaviors such as physical activity (PA), eating fresh foods and hand washing [38]. Another cross-sectional study to assess dietary intake of adults 18 years and above in Indonesia during the period of the lockdown showed more respondents eating out less, and more using supplements [39]. However, in another COVID-19 and diet study conducted during the period of the LSSR in Indonesia, authors found that more than 50% of respondents were not following good nutritional behavior (defined as home cooked food, fresh food, adequate water intake) [40]. A review of dietary pattern changes during the first wave of the COVID-19 pandemic across 18 countries including Indonesia also revealed a marked increase in the consumption of refined carbohydrate foods such as pastries, breads and pies, and raised concerns about the health fallouts due to these shifts in the long-term, post-COVID-19 [41]. Our findings provide context and clarity to some of these studies by hypothesizing that although COVID-19 brought increased awareness and adherence to health behaviors, these gains were not sustained beyond the period of social restrictions.

Studies investigating PA behaviors related to the COVID-19 pandemic have yielded inconclusive results [17,42]. Syafiq et al. (2022) found that PA behaviors were about even in an Indonesian sample of respondents, with 52% reporting no PA [17]. Amalia et al., 2021 surveyed 83 individuals from 32 cities in Indonesia to assess PA behaviors during the COVID confinement period [42]. They reported that most respondents had moderate activity levels with females more active than males. A higher intensity of working from home negatively correlated with a physical activity score, however, it was not statistically significant [42]. Our study also revealed inconclusive findings on PA. Since PA is related to the built environment, having many public parks closed during the pandemic may have led to less people engaging in leisure time PA.

At the interpersonal level, one major theme on the impact of the COVID-19 pandemic on dietary behavior of households emerged. Our informants indicated that COVID-19 did not bring significant improvement to family meal patterns and dietary behaviors. On the contrary, their views implied that unhealthy eating patterns of families may have surpassed the pre-COVID-19 period. Although lockdowns meant more meals taken at home, these were not necessarily home-cooked meals. There is consistent evidence that higher consumption of away from home meals compared to home-cooked meals increases the risk for diet-related chronic diseases [43,44,45]. Informants also highlighted sedentary behaviors and unhealthy eating patterns increasing among children due to school closure and more screen time at home. These perspectives are supported by other studies on the consumption of away-from-home foods during the COVID-19 pandemic in Indonesia. In a survey on dietary behaviors of college students in Indonesia studying from home during COVID-19, authors found an increase in the option of buying cooked food from online delivery services [46]. Another quantitative study among college students in Indonesia showed no significant decrease in consumption of packaged foods and sugar sweetened beverages during the pandemic in August 2020 compared to December 2019 [21].

At the community level, one theme related to the food environment since COVID-19 emerged. Informants suggested that while the pandemic did not negatively impact the availability of healthy foods generally, the availability of perishable foods such as fruits and vegetables may be negatively impacted by lack of proper facilities for preservation when transporting these foods along the supply chain. Our informants noted that Indonesia may benefit from national level policies to promote the preservation and conservation of perishable foods. Informants felt that the pandemic also did not impact access to healthy food types because with the proliferation of online food retail platforms, people still had access to healthy foods. This perspective is supported by current studies on food systems resilience during the pandemic in Indonesia. In a qualitative study, Paganini and colleagues described the perspectives of small holder farmers in Indonesia during the period of government restriction policies in Indonesia from March to June 2020 [47]. They found that compared to farmers in South Africa and Zimbabwe, the local food systems in Indonesia had higher social capital and adaptive capabilities, and continued to operate during pandemic restrictions because restrictions were enforced at grass-root level [47]. On the other hand, we found that through decreased purchasing power from job losses and business failures, affordability was an issue. This is in line with findings of a cross-sectional survey among adults in Indonesia during COVID-19 confinement where respondents reported that increase in the price of fruits and vegetables affected their consumption of these items [48]. Informants reported that the government provides cash transfers to poor families, however, it is often inadequate, and it is not clear whether people use the cash for food. Our experts implied that decreased purchasing power and high cost of foods may have increased food insecurity and malnutrition. This also concurs with the results of Syafiq et al., that showed that COVID-19 negatively impacted the food security of families equally in both urban and semi-urban areas [17].

At the environmental level we appraised the impacts of the COVID-19 pandemic on marine pollution. Indonesia, an island country with a larger proportion of its surface area made up of ocean, has one of the highest levels of marine pollution in the world [26]. Our findings suggest that pollution levels increased even more in Indonesia because of COVID-19. Our informants furnished many reasons for this. They described an increase in medical and hygienic wastes from masks and PPEs, as well as from plastic packaging from online food orders that get dumped into the seas. Additionally, they noted that the government prioritizing the health sector during COVID-19 meant less attention given to addressing marine pollution. Some environmental researchers reported that community-led programs to reduce marine waste were reduced due to restriction of mobility in the early phase. These perspectives reflect the findings of other studies on the impacts of the COVID-19 pandemic on marine environments globally [30], and specifically for Indonesia [31]. While promoting the wearing of masks and PPE was aimed at reducing infections and mortality due to COVID-19, however, more pollution of the oceans and seas with medical waste will likely further affect fish quality and adversely impact the health of the general population in the long term.

This empirical research has some limitations which we addressed as much as possible in order to improve the validity of our approach. We interviewed researchers and health professionals, a greater proportion of whom are doctoral degree holders, with expertise in relevant fields to provide their perspectives on the impacts of the COVID-19 pandemic on diets, health and the marine environment. We acknowledge that each of these informants would have their own biases and that their perspectives may not accurately represent the perspectives and/or realities of the general Indonesian population with a lower education level. However, purposively recruiting a variety of experts, in order to get a blend of perspectives on the different topics addressed, was also part of our study process to attain validity. Additionally, we recruited an adequate sample size for each category of experts to ensure that we achieve thematic saturation, which we did. We also shared our finding with study collaborators from Indonesia, who were not interviewed, to corroborate our findings. Nevertheless, due to the expert interview nature of this work, this study was not able to tap into detailed coping mechanisms and innovations in different socioeconomic levels. Future qualitative works using focus groups and ethnographic approaches may consider recruiting respondents from a more diverse population to better understand the citizens’ lived experience.

Further, from the environmental point of view, our informants’ perspectives were focused on the impacts of the COVID-19 on marine pollution. Other stakeholders at the grass root level, such as farmers and fishermen, may have other perspectives beyond the scope covered in this study that can be addressed in future studies.

Moreover, fluency in English was an inclusion criterion to participate in the study. While many of our participants are highly educated and have enough fluency in English to participate in the study, we anticipated that an impromptu expressing of their thoughts and ideas in a second language might still prove a challenge. To address this and ensure that our participants present their perspectives as clearly and in great detail as possible, we sent the interview questions to them ahead of time, so they could provide clear and well reflected perspectives. Both their written and verbal responses were analyzed as part of our findings. Finally, the interviews were conducted remotely, online. Although the interviews were conducted with video on, and interviewers took note of the non-verbal cues from participants as much as possible, we acknowledge that we may have missed out on other non-verbal cues of in-person meetings, that could have provided a richer meaning and context to our findings than captured in the more static online environment.

The socio-ecological framework employed to develop the interview question guide and analyze the themes allowed us to cover a broad scope of topics relating to COVID-19 impacts along each level of the framework. Additionally, by recruiting experts from different fields, we gained in-depth insights based on multi-disciplinary perspectives on the different factors associated with the COVID impacts on the Indonesian population. Finally, the framework used will facilitate better targeting of interventions and programs to meet the needs of specific vulnerable sub-population at each level.

### Implications for Policy and Directions for Additional Research

This study leverages the richness of perspectives from a multi-disciplinary group of researchers and health professionals to offer some provisional policy recommendations and suggestions for future research.

Our results suggesting a lack of continuity of adherence to health behaviors beyond the social restrictions period may be a pointer to the need for environmental supports to undergird behavior change programs. Previous studies indicate that the context of individuals, including the social media environment and advertising exert strong influence on health behaviors [24,49]. Informants pointed to the avalanche of health information, both authentic and non-authentic through social media during the pandemic. They also reported more screen time for children due to school closure, which may potentially indicate higher exposure to television advertising. If no action is taken by the government to curb the influence of social media and advertising, they may potentially contribute to unhealthy food choices among individuals and families, and increase the burden of diet-related chronic illnesses in the general population. Therefore, Indonesia may benefit from stricter social media and advertising policies. We also recommend more empirical studies that combine qualitative and quantitative methods to further investigate the role of the social media and adverting on awareness and adherence to health behaviors in the post-COVID-19 era in Indonesia. Further, more studies assessing the long-term impacts of COVID-19 on family eating patterns are warranted to help inform the design of dietary interventions targeting households in Indonesia.

At the regional and community level, informants raised the issue of food preservation, particularly of perishable food types such as fruits and vegetables, along the supply chain and suggested the need for better food preservation policy. We echo this recommendation, as it will help to reduce food waste and reduce the problem of food insecurity among vulnerable sub-populations. Our results also suggest that the contents of government food aid packages to low-income households put in place during the pandemic may need to be reviewed to include more healthy food options such as fruits, vegetables and legumes, which are not affordable for low-income populations. However, for this to be beneficial, the problem of food preservation will need to be addressed first. It is likely that the decision on what to include in food-aid packages are based more on expediency than on nutritional benefits. However, providing more carbohydrate foods to low-income families will likely drive up the obesity epidemic in the long-term. Thus, addressing the food preservation policy may prove to be more cost effective in terms of health benefits of more availability and access to fruits and vegetables for poorer families. This also is an area of further research. More qualitative and quantitative studies addressing cost effectiveness of food preservation along the supply chain from rural to urban centers on population health outcomes are warranted for Indonesia.

Our findings on the substantial additional increase in marine pollution related to hygiene behaviors promoted during the pandemic suggests an unintended negative consequence of government policy to promote population health during a pandemic. Further, the indication by informants that the government prioritized the health sector, during the pandemic, also implies that the already inadequate waste management programs received even less attention. This would foment a two-fold setback on the marine pollution scenario for Indonesia, and underscores the need for urgent action by the Indonesian government to better prioritize waste management policies going forward. Here too, more quantitative studies with longitudinal designs are needed to further investigate the long-term impacts of COVID-19 related wastes on marine species and human health in Indonesia.

## 6. Conclusions

Our findings reveal that the increased awareness and adherence to health behaviors observed during the active phase of the COVID-19 pandemic appear to be transient. Additionally, the pandemic may have contributed to higher rates of food insecurity and obesity, thus adding to the double burden of disease paradox. Higher levels of marine pollution attributed to COVID-19 related hygiene protocols suggest an unintended negative consequence of government policies to improve population health during a pandemic. Insights from our study may be useful for policy makers and health and nutrition professionals in Indonesia to build on the gains from heightened health awareness of the active phase of the COVID-19 pandemic by formulating policies and developing interventions that are geared towards sustainable changes in health behaviors among the Indonesian population. However, more empirical studies, both quantitative and qualitative, are needed to further corroborate our findings and inform policies and programs to mitigate the long-term impacts of the pandemic on the diets, health and marine pollution in Indonesia.

## Figures and Tables

**Table 1:** COVID-19 cases, deaths and vaccination rates in S.E. Asia region (25 June 2022).

Country	Total Population	Confirmed Cases ^1^	Total Deaths ^2^	Fully Vaccinated (%)
Philippines	109,850,251	3,699,251	60,507	64.11
Indonesia	274,061,093	6,076,894	156,711	61.51
Singapore	5,858,949	1,397,074	1408	85.35
Malaysia	32,422,628	4,552,359	35,745	84.09
Thailand	69,833,165	4,509,541	30,559	75.59
Vietnam	97,501,966	10,742,234	43,084	81.97
Myanmar	54,478,228	NA	NA	NA
Cambodia	16,761,610	136,262	3056	85.78
Brunei	438,259	159,591	225	97.55
Laos	7,294,985	210,233	757	69.78
Timor-Leste	1,445,006	22,949	133	NA

Notes: ^1^ Actual number of cases may be higher than confirmed cases due to limited testing. ^2^ Actual number of deaths may be higher than reported due to limited testing and challenges in determining the cause of deaths. Source: https://coronavirus.jhu.edu/region [3].

**Table 2:** COVID-19 Related Questions for Study Informants.

**Icebreaker Question:** If you could eat only one food for the rest of your life, what would it be? And what is it about this food that you like? (Or, “What is your preferred main-meal time: breakfast, lunch, dinner and why?) **Section 1: Individual-level Factors** First, let’s talk about general awareness about diet and health among the Indonesian population 1.1. What are some of their food preferences/food choices that may impact their health, especially increasing risk for obesity, high blood pressure and high cholesterol? 1.2. Where do people typically get advice on diet and health? 1.3. What are the current recommendations for a healthy diet from the Indonesian dietary guidelines? 1.3.1. Are most people aware of the Indonesian dietary guidelines? 1.3.2. Do they typically follow these guidelines? 1.3.3. In your opinion, what gets in the way of adhering to the guidelines? In what ways, if any, has this changed since the COVID-19? **Section 2: Family/Interpersonal Level Factors** Tell me about the usual eating and meal patterns of most families prior to COVID-19 (e.g., communal meals vs. individual meals; one big meal vs. multiple meals; regional and ethnic differences in meal patterns) 1.1. In what ways have the effect of lifestyle changes in the past 3 or 4 decades impacted family eating patterns 1.2. What about social media and advertisement, etc. (if not already addressed in 1.2.1 above)? How have eating and meal patterns changed since COVID-19? Next, let’s talk about eating at home versus eating outside 3.1. What are your thoughts on fast food or restaurant food consumption In Indonesia? 3.1.1. What prompt people to eat out (including ordering food online, take out or home delivery), rather than cook/eat at home? 3.1.2. In your opinion, how does this habit influence food choices/preferences and consumption patterns (including snack food, and beverage)? 3.2. What gets in the way of cooking at home? (Ask, only if not already addressed above) How has this changed since COVID-19 if at all? **Section 3: Challenges to access, availability and affordability of healthy foods.** In this section, I’ll ask about availability, access, and affordability of foods that are generally considered healthy. **1.** **Availability** 1.1. Prior to the outbreak of the COVID-19 pandemic, where do people typically buy the food they eat? [Probes: What types of food retail outlets are typically available in most regions of Indonesia: supermarkets, grocery stores, open markets, convenience stores?] 1.2. Comment on the availability of foods that are generally considered healthy, such as fruits, vegetables, whole grains, legumes and nuts in food retail outlets. 1.2.1. Comment on the availability of foods that are generally considered healthy, such as fruits, vegetables, whole grains, legumes and nuts in food retail outlets. 1.2.2. Is there a variety of such foods available in the retail outlets? 1.3. Describe urban versus rural differences in the availability of fruits, vegetables, whole grains, legumes, and nuts? 2. In what ways, if any, has the availability of these types of healthy foods changed since COVID-19? 3. Now, let’s talk about the availability of fish 3.1. What types of fish are readily available (fresh, frozen, dried, salted, etc.)? 3.2. Please describe regional differences in the availability of fish (e.g., urban vs. rural, coastal vs. non-coastal, mountainous regions, if not already addressed above) 4. In what ways, if any, have these changed since the COVID-19 pandemic? **5.** **Access (*Now I would like to ask you about how far or near the food retail outlets are to consumers*)** 5.1. Describe the access of these food retail outlets to most consumers, especially in low-income neighborhoods. For instance, prior to the COVID-19 pandemic, are retail outlets that sell healthy food types located near low- income neighborhoods? 5.2. What mode of transportation is readily available between the food retail outlets and low- income neighborhoods? 6. How has the COVID-19 impacted access to healthy foods such as fruits, vegetables, whole grains, legumes and nuts? What about access to fresh fish? 6.1. In what ways, if any has the COVID-19 affected access to healthy foods for needy population subgroups, e.g., people needing food assistance such as the poor, the sick, or the elderly? 6.2. In what ways, if any, has the COVID-19 impacted government and /or community programs resources for needy populations **7.** **Affordability** 7.1. Comment on the affordability of foods that are generally considered healthy (like fruits, vegetables, whole grains, legumes, nuts and fish), especially for low- income households. Are these food options relatively cheap? 7.2. What emergency resources are available to low-income households (probes: food pantries, soup kitchens, food banks, and other community-based food distribution programs) in the event that they do not have enough money to purchase healthy food through normal channels? 8. In what ways, if any has the COVID-19 affected affordability of these healthy foods? **Section 4: Impacts of marine pollution on fish quality and availability** Now we will discuss about the issue of environmental pollution and its impact on the quality and availability of fish and other healthy foods in Indonesia In what ways, if any, does marine pollution impact fish quality 1.1. What about fish availability? 1.2. In what ways, if any, does marine pollution affect the availability and quality of other healthy food types such as fruits and vegetables, whole grains and nuts? 1.3. As an expert in the field of …, what other factors beyond nutrition comes to mind, that might have adverse effects on the health of Indonesians, that we have not touched upon? In what ways if any, has the COVID-19 impacted marine pollution in Indonesia In what ways if any, has the COVID-19 impacted other types of pollution in Indonesia 3.1. Now, let’s discuss government policy on pollution reduction 3.2. Tell me about the current policies in place to reduce marine pollution in Indonesia that you’re aware of 3.3. What major barriers have there been, if any, to implementing these policies? 3.4. In your opinion, what can the government do differently? In what ways, if any, has the COVID-19 impacted government policy for waste disposal and management?

**Table 3:** Demographic Characteristics of Study Informants.

	Total (*n* = 27)	Nutrition/Public Health Researcher (*n* = 10)	Healthcare Provider (*n* = 8)	Environmental Researcher (*n* = 9)
**Age (Year) mean, (SD)**	**46.1 (11.9)**	48.5 (12.1)	38.7 (14.1)	49.3 (7.7)
**Female *n*, (%)**	**19 (70.4)**	9 (90.0)	7 (87.5)	3 (33.3)
**Ethnicity *n*, (%)**				
Javanese	**14 (51.9)**	6 (60)	2 (25.0)	6 (66.7)
Sundanese	**2 (7.4)**	2 (20)	0 (0.0)	0 (0.0)
Batak	**2 (7.4)**	0 (0.0)	2 (25.0)	0 (0.0)
Buginese	**2 (7.4)**	1 (10.0)	1 (12.5)	0 (0.0)
Sulawesi	**2 (7.4)**	1 (10.0)	0 (0.0)	1 (11.1)
Lampungenese	**1 (3.7)**	0 (0.0)	1 (12.5)	0 (0.0)
Chinese-Indonesian	**1(3.7)**	0 (0.0)	0 (0.0)	1 (11.1)
Other	**3(11.1)**	0 (0.0)	2 (25.0)	1 (11.1)
**Highest level of education *n*, (%)**				
Bachelor’s Degree	**3 (11.1)**	0 (0.0)	2 (25.0)	1 (11.1)
Masters’ Degree	**5 (18.5)**	2 (20.0)	0 (0.0)	3 (33.3)
Medical Practitioner/Clinical Nutritionist	**6 (22.2)**	0 (0.0)	6 (75.0)	0 (0.0)
Doctoral Degree (Ph.D.)	**13 (48.2)**	8(80.0)	0 (0.0)	5(55.6)
**Years of Work Experience *n*, (%)**				
Less than 5 years	**6 (22.2)**	1(10.0)	4 (50.0)	1(11.1)
5 to 10 years	**3 (11.1)**	1 (10.0)	2 (25.0)	0 (0.0)
11 to 20 years	**9 (33.3)**	4 (40.0)	1 (12.5)	4 (44.4)
More than 20 years	**9 (33.3)**	4 (40.0)	1 (12.5)	4 (44.4)

**Table 4:** Summary of Findings.

Socio-Ecological Framework Level	Theme/Sub-Theme	Key Findings
Individual	COVID-19 impacts on awareness about and adherence to health behaviors	Higher awareness and adherence to healthy life-style Higher consumption of foods that boost immunity Telemedicine services increased Avalanche of health information on social media from authentic and non-authentic sources Health behavior change not sustained
Family/Interpersonal	COVID-19 impacts on family meal patterns and dietary behaviors	Many restaurants closed, but more online options More eating from home during the lockdown, but not clear if home-cooked or ordered online More snacking behavior Cooking practices, unhealthy eating patterns soon rebounded after social restrictions ended More screen time and exposure to TV advertising for children and adolescents meant more demand for away-from-home foods
Community/Regional	COVID-19 impacts on availability, affordability and access to healthy foods: COVID-19 impacts on availability of healthy foods	Temporary shortage of essential food commodities due to panic purchase at the beginning of the pandemic Disrupted supply chain during mobility restrictions affected the availability of fruits and vegetables Preserving fresh produce along the food supply chain is problematic Healthy food availability supplemented by home gardening and frozen foods
	COVID-19 impacts on availability, affordability and access to healthy foods: COVID-19 impacts on affordability of healthy foods	Decreased purchasing power and high cost of foods may have increased food insecurity and malnutrition Government aid programs to support the poor generally in-sufficient but often supplemented by food aid programs from charitable organizations
	COVID-19 impacts on availability, affordability and access to healthy foods: COVID-19 impacts on access to healthy foods	No negative impacts on food access More presence of online retail platforms may have drove down the price of some food items
Environmental	COVID-19 impacts on marine pollution	Overall increase in marine pollution due to: More medical and hygienic wastes dumped Plastic packaging from online food orders Government prioritizes other projects than waste management Community programs to reduce marine waste were affected

## Abbreviations

COVID-19	Corona Virus Disease 2019
DIY	Daerah Istimewa Yogyakarta
IRB	Institutional Review Board
LSSR	Large-Scale Social Restrictions
MetS	Metabolic Syndrome
PA	Physical Activity
PPE	Personal Protective Equipment
SARS-CoV-2	Novel Corona Virus
SEM	Socio-ecological model
SES	Socioeconomic Status
UNDIP	Universitas Diponegoro
WHO	The World Health Organization
